# Clinical and neuroimaging differences between posterior cortical atrophy and typical amnestic Alzheimer’s disease patients at an early disease stage

**DOI:** 10.1038/srep29372

**Published:** 2016-07-05

**Authors:** Guoping Peng, Jianqin Wang, Zhan Feng, Ping Liu, Yafei Zhang, Fangping He, Zhongqin Chen, Kui Zhao, Benyan Luo

**Affiliations:** 1 Department of Neurology, First Affiliated Hospital, Zhejiang University School of Medicine, Hangzhou, China; 2 Laboratory of Brain Medical Center, First Affiliated Hospital, Zhejiang University School of Medicine, Hangzhou, China; 3 Department of Radiology, First Affiliated Hospital, Zhejiang University School of Medicine, Hangzhou, China; 4 Department of PET Center, First Affiliated Hospital, Zhejiang University School of Medicine, Hangzhou, China

## Abstract

To identify clinical and neuroimaging characteristics between posterior cortical atrophy (PCA) and typical amnestic Alzheimer’s disease (tAD) patients at an early disease stage, 16 PCA and 13 age-matched tAD patients were enrolled. Compared with tAD patients, PCA patients showed higher mean recognition and recall test scores, and lower mean calculation, spatial attention, shape discrimination, and writing test scores. Mean right hippocampal volume was larger in PCA patients compared with tAD patients, while cortical gray matter (GM) volume of bilateral parietal and occipital lobes was smaller in PCA patients. Further, when compared with tAD patients, significant hypometabolism was observed in bilateral parietal and occipital lobes, particularly the right occipitotemporal junction in PCA patients. Additionally, there were significant positive correlations in recognition and recall scores with hippocampal volumes. In PCA patients, calculation and visuospatial ability scores are positively associated with GM volume of parietal and occipital lobes. And only spatial attention and shape discrimination scores are positively associated with regional glucose metabolism of parietal and occipital lobes. Therefore, PCA patients display better recognition and recall scores, which are associated with larger hippocampal volumes and poorer performance in visual spatial tasks because of marked GM atrophy and hypometabolism of parietal and occipital lobes.

Alzheimer’s disease (AD) is a progressive neurodegenerative disease that typically displays memory deficits^1^, but can also exhibit non-amnestic syndromes including posterior cortical atrophy (PCA)^2^, logopenic variant primary progressive aphasia^3^, and frontal variant AD^4^.

PCA is clinically characterized by a progressive decline in visual processing, literacy, numeracy, and other functions dependent on parietal, occipital, and occipitotemporal brain regions^2^,^5^. Indeed, visual cognitive deficits may be more prominent than memory, language, and other cognitive abnormalities^6^. However, very few studies have been performed in Chinese populations, particularly for patients at an early disease stage. PCA is now recognized in AD diagnostic and research criteria as the most common atypical AD phenotype^7^. Earlier studies show that PCA subjects display similar patterns of cerebrospinal fluid (CSF) and amyloid β (Aβ) neuroimaging biomarkers as typical amnestic AD (tAD) patients, suggesting an underlying AD neuropathology in PCA^8^,^9^. Nevertheless, some PCA patients display other neuropathological changes, including corticobasal degeneration (CBD), dementia with Lewy bodies (DLB), and Huntington’s or Creutzfeldt–Jakob disease (CJD)^10^,^11^.

Diagnosis of PCA depends on core clinical features, which is further supported by neuroimaging and neuropathological evidence^12^. Cohort studies performed in non-Chinese Caucasian populations showed bilateral occipitoparietal and occipitotemporal atrophy with right-sided predominance in PCA patients^13^. Medial temporal lobe atrophy is an early feature in tAD, yet atrophy is also detected in posterior areas such as the precuneus and posterior cingulate gyrus^14^. Compared with tAD patients, PCA patients show greater atrophy in the right occipitotemporal cortex^15^. Moreover, voxel-based image analysis of ^18^F-fluoro-deoxyglucose-PET (FDG-PET) in tAD patients suggests the earliest hypometabolic regions occurs in the posterior cingulate cortex and mesial temporal structures^16^. In contrast, the initial hypometabolic region in PCA patients is located in the primary visual cortex and the visual associated cortices^3^. Several studies showed cerebral atrophy and neuropsychological deficits in the expected brain regions and cognitive domains^15^,^17^,^18^. Nonetheless, the difference of neurocognitive and neuroimaging characteristic between PCA and tAD patients in Chinese population is largely unknown, and also for the correlation between the neuropsychological tests performance and the atrophic or hypometabolic regions in these patients^19^.

Here we examined 16 PCA patients that first presented with progressive visual disorders, and 13 tAD patients that presented with episodic memory loss, all of whom are at a comparatively early disease stage. Differences in clinical manifestations, biomarker levels, regional atrophy, and glucose metabolism between the groups were compared, with the possible relationship between neuropsychological tests performance and neuroimaging characteristics in PCA patients further analyzed.

## Methods

### Subjects

Sixteen PCA and 13 tAD patients were recruited from the Memory Clinic and Neurology Unit, First Affiliated Hospital of Zhejiang University, Hangzhou, China. All patients underwent a detailed clinical history interview and physical examination, neuropsychological assessment, and a T1-3D MRI scan. The majority (23/29) of patients underwent ^18^F-FDG-PET scanning.

Participants with PCA fulfilled previously proposed clinical diagnostic criteria^19^. Core clinical features included: insidious onset and gradual progression; presentation of visual complaints in the absence of a significant primary ocular disease; relative memory preservation and early insight into the disorder; disabling visual impairment throughout the disorder; and absence of stroke or tumor. Supportive features included: alexia, ideomotor apraxia, agraphia, acalculia, onset before 65 years old. Patients were excluded if they exhibited early symptoms of myoclonus, extrapyramidal motor signs, and evident hallucinations, which indicate an underlying CBD or DLB pathology. Furthermore, diagnosis of PCA was supported by follow-up visits (mean follow-up 19 ± 9 months).

Participants with tAD showed onset of an episodic memory deficit, and fulfilled the National Institute of Aging-Alzheimer’s Association (NIA-AA) criteria for probable AD^20^. And fifteen healthy, age-, education-, and sex-matched control subjects (Clinical Dementia Rating [CDR] of 0) were also recruited.

Informed consent was obtained from all participants or their assigned surrogate decision makers. The study was approved by the Committee of First Affiliated Hospital of Zhejiang University on Human Clinical Research. All experiments were performed in accordance with relevant guidelines and regulations.

### AD biomarkers and apolipoprotein E 4 (*APOE4*) allele analysis

Nine subjects (including five PCA and four tAD patients) underwent lumbar punctures in the morning. Cerebrospinal fluid (CSF) was centrifuged at 1800 × g for 10 min at 4 °C, then transferred to new polypropylene tubes, and stored at −20 °C until biomarker analysis. Serum specimens were collected from all patients and stored at −20 °C before biomarker analysis. Phosphorylated tau (p-tau), total tau (t-tau), and Aβ1–42 concentrations were analyzed by ELISA (CSF specimens: INNOTEST; Innogenetics, Ghent, Belgium, and serum specimens: Arigobio, Hsinchu, Taiwan, ROC). Using reference ranges, CSF profiles are considered to be a highly sensitive indicator of a clinical diagnosis of AD when tau >350 pg/mL and Aβ1–42 < 500 pg/mL. Genomic DNA was also collected and the *APOE4* allele was analyzed in all patients.

### Neuropsychological assessment

All participants underwent a general neuropsychological assessment at the day of MR scan, including the Mini-Mental State Examination (MMSE) and CDR. Further, a battery of neuropsychological tests designed to assess memory, attention, calculation, executive function, visual-spatial ability, and language function were also used. Moreover, in all patients, components of Gerstmann and Bàlint–Homes’ syndromes were assessed using unstandardized tasks during neurological and/or neuropsychological evaluation.

### Structural MRI and data processing

T1-weighted volumetric MRI brain scans were performed using a 3.0T scanner (General Electric Medical System, Atlanta, GA, USA) with a spoiled gradient recalled sequence. Left and right hippocampal volume measurements were acquired and calculated as previously described^21^. For cortical GM volume comparisons, standard methods of optimized voxel-based morphometry (VBM) procedures^22^ within the VBM8 toolbox of statistical parametric mapping version 8 (SPM8, http://www.fil.ion.ucl.ac.uk/spm) were used. Diffeomorphic Anatomical Registration through Exponentiated Lie algebra (DARTEL) was used to generate a study-specific template by non-linearly aligning GM images to a common space^23^. Native GM and white matter images were spatially normalized to the DARTEL template using individual flow fields. Images were smoothed using an 8-mm full-width at half maximum isotropic Gaussian kernel. To identify potential outliers, final smoothed-modulated-warped GM images were checked for sample homogeneity using the VBM8 toolbox. The implicit best-fit mask was used for statistical analysis, and provided an optimal balance between noise reduction and preservation of actual GM. Group comparisons were made on a voxel-level using two-sample *t*-tests. The significance threshold was false discovery rate (FDR)-corrected *p* < 0.05.

Standardized regions of interest (ROIs) were defined on a MRI template image representing the brain anatomy, and in accordance with the Montreal Neurological Institute (McGill University, Montreal, QC, Canada) brain atlas. The following ROIs were formed by merging and pooling subsets from the original Automated Anatomic Labeling atlas^24^: temporal lobe (T1: superior temporal gyrus, T2: middle temporal gyrus, T3: inferior temporal gyrus, HES: Heschl gyrus), parietal lobe (P1: superior parietal gyrus, P2: inferior parietal gyrus, AG: angular gyrus, SMG: supramarginal gyrus), occipital lobe (O1: superior occipital gyrus, O2: middle occipital gyrus, O3: inferior occipital gyrus, Q: cuneus, V1: calcarine fissure and surrounding cortex, LING: lingual gyrus, FUSI: fusiform gyrus), posterior cingulate gyrus (PCIN) and precuneus (PQ). This set of ROIs was chosen to reflect the raters’ standard clinical diagnostic practice and our GM findings. Correlations between the neuropsychological scores and mean GM volume of ROIs in PCA patients were analyzed with a statistical threshold of *p* < 0.05.

### ^18^F-FDG-PET scan and data processing

PET images were acquired using a PET/CT scanner (Siemens Biograph Sensation 16, Munich, Germany) in 3D scanning mode. Forty-five minutes after an intravenous bolus injection of ^18^F-FDG (5.5–7.4 MBq/kg of body weight), after at least 6 h of fasting, some patients (tAD, *n* = 11; PCA, *n* = 12) underwent a 10-min emission examination. Before statistical analysis, all images were spatially normalized to the Montreal Neurological Institute space to correct inter-subject anatomical variability. Global metabolism was normalized using proportional scaling by the count of each voxel to the total brain count. Normalized images were smoothed by convolution using an isotropic Gaussian kernel with a 12-mm full-width at half maximum to accommodate inter-subject differences in gyral and functional anatomies, and increase the dataset signal-to-noise ratio. Spatial preprocessing and statistical analyses were performed using SPM8 software and Matlab2010b for Windows (Mathworks, Natick, MA, USA). Brain metabolism comparisons were performed on a voxel-by-voxel basis using two-sample *t*-tests. Using an extent threshold of 50 voxels, regions reaching uncorrected *p* < 0.001 were considered statistically significant.

The following ROIs were also generated: inferior parietal lobe, superior parietal lobe, superior temporal gyrus, middle frontal gyrus, posterior cingulate cortex and precuneus, and occipital lobe. Within ROIs, correlations between neuropsychological scores and mean regional cerebral glucose metabolism (rCMg) in PCA patients were examined using a statistical threshold of *p* < 0.05.

### Statistical analysis

Values were expressed as mean ± S.D, or as frequencies. Demographic data (age, education years, and neuropsychological scores) between groups were assessed using analysis of variance. Comparisons between study groups, including sex differences and prevalence of clinical characteristics, were assessed using chi-square tests. Correlations between neuropsychological scores and hippocampal volume, GM volume, and rCMg of ROIs were analyzed using Spearman’s correlation test. All analyses were performed using SPSS 19.0 (SPSS Inc., Chicago, IL, USA), with *p* values of 0.05 indicating significant differences.

## Results

### Demographic characteristic between different groups

Demographic information and the frequency of cerebrovascular risk factors for 16 PCA patients, 13 tAD patients, and 15 healthy controls are presented in Table 1. On average, both patient groups showed mild to moderate dementia severity (mean MMSE: 19.1 ± 2.8 *vs* 18.9 ± 2.5, *p* = 0.843; mean CDR: 1.93 ± 0.68 *vs* 1.92 ± 0.64, *p* = 0.968), and comparatively early disease stages (mean disease duration: 2.4 ± 0.9 *vs* 3.0 ± 1.0, *p* = 0.101). Chi-square test and analysis of variance found no significant differences in sex, age, and years of education, and all patients were right-handed. Additionally, there were no significant differences in frequency of each vascular risk factor between groups.

### *APOE4* allele frequency and AD biomarker profiles in PCA and tAD patients

**Figure 1:**
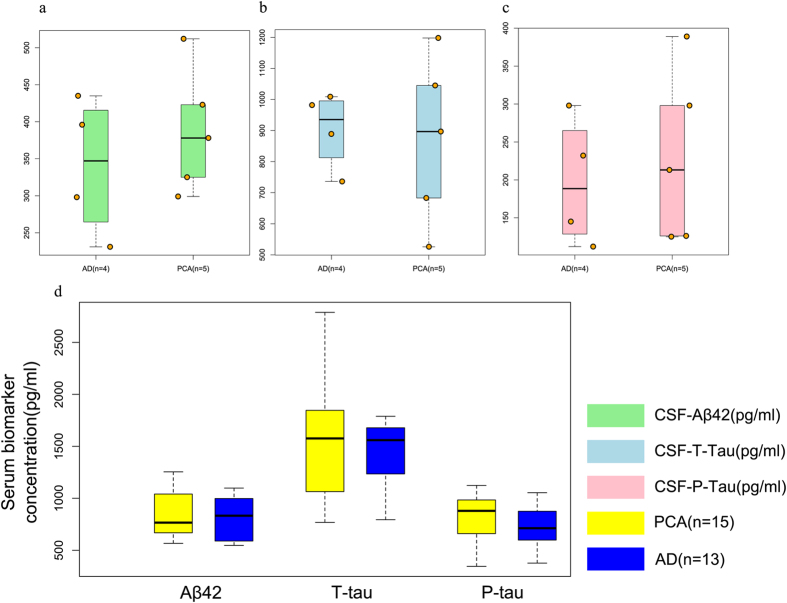
The concentration of AD biomarkers including Aβ1–42, t-tau and p-tau. (**a**) The difference of mean CSF Aβ1–42, t-tau and p-tau levels in PCA patients and tAD patients. (**b**) The difference of mean serum Aβ1–42, t-tau and p-tau levels in PCA patients and tAD patients. t-tau, total tau; p-tau, phosphorylated tau.

*APOE4* has been reported to be a risk factor of AD. In our study, we did not found significant difference in *APOE4* allele frequency between the PCA and tAD groups, with two patients in each group being ɛ4 homozygous (Table 1). Moreover, one PCA patient and two tAD patients had familial AD history and these proband were received genetic testing. There is no early onset AD related genetic mutation in the PCA patient while two tAD patients with an amyloid precursor protein (APP) gene site mutation (data not shown). Five PCA patients underwent CSF biomarker examination, and showed similar profiles, including Aβ1–42, t-tau, and p-tau concentrations, as tAD patients (Fig. 1a). All four tAD patients had a decreased Aβ1–42/t-tau ratio < 0.5. The majority (4/5) of PCA patients also had low Aβ1–42 concentrations, and raised tau-to-Aβ1–42/t-tau ratio < 0.5. Mean serum Aβ1–42, t-tau, and p-tau levels did not differ between both groups (Fig. 1b).

### Clinical symptoms and cognitive assessment scores in PCA and tAD patients

During diagnosis, no tAD patients reported visual complaints or severe dressing apraxia, while all the 16 PCA patients had a different range of visuoperceptual and/or visuospatial impairments. As shown in Table 2, PCA patients had a much higher frequency of dressing apraxia (*p* = 0.013), construction apraxia (*p* = 0.027), finger agnosia (*p* = 0.000), prosopagnosia (*p* = 0.000), and symptoms of Bàlint–Homes’ and Gerstmann syndromes. Patients in the PCA and tAD groups showed distinct cognitive performances (Table 3): e.g., tAD patients performed better in the clock drawing test (CDT) (*p* = 0.001), calculation, writing, spatial attention, and shape discrimination than PCA patients (all *p* = 0.000), while two memory measures were significantly less impaired in PCA patients (short recognition memory of words, and California verbal learning test, CVLT of 10-min recall; both *p* = 0.000). However, digit span, naming, and lexical fluency scores were similar between both groups. Moreover, PCA patients had a higher mean Hamilton Anxiety Rating Scale score than tAD patients (*p* = 0.000).

### Mean hippocampal volumes in PCA and tAD patients

**Figure 2:**
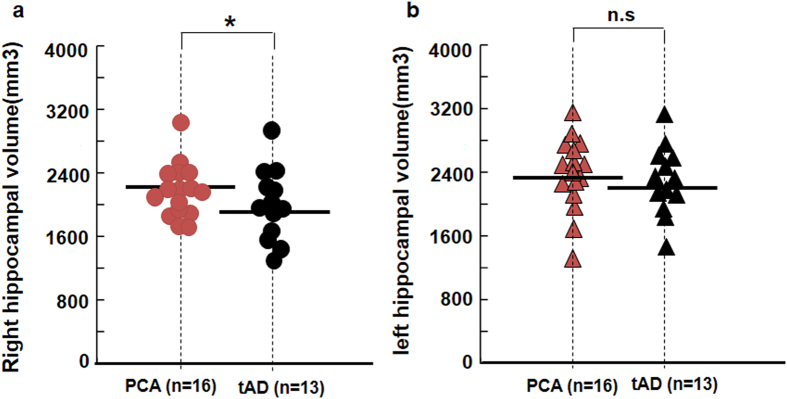
The difference of mean hippocampal volumes between PCA and tAD groups. (**a**) Scatterplot of individual relative right hippocampal volume in tAD and PCA patients. (**b**) Scatterplot of individual relative left hippocampal volume in tAD and PCA patients. *p < 0.05, compared with the tAD group; ns, not significant.

**Figure 3:**
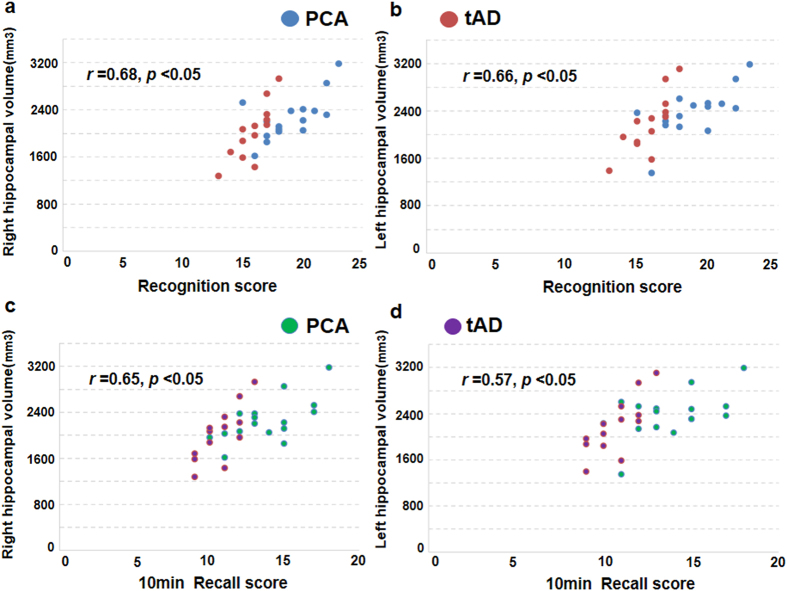
The correlation between hippocampal volume and neurocognitive scores. (**a**) The mean right hippocampal volume was positively correlated with recognition score in both tAD and PCA groups. (**b**) The mean left hippocampal volume was positively correlated with recognition score in both tAD and PCA groups. (**c**) The mean right hippocampal volume was positively correlated with short-time recall score in both tAD and PCA groups. (**d**) The mean left hippocampal volume was positively correlated with short-time recall score in both tAD and PCA groups. Using the Spearman correlation test with the significant difference of *p* < 0.05.

After normalization, right hippocampal volume in the tAD group was significantly smaller compared with that in the PCA group (2024 ± 412 mm^3^ *vs* 2262 ± 347 mm^3^, *p* = 0.041, Fig. 2a). Interestingly, left hippocampal volume in the tAD group was slightly but insignificant smaller than that in the PCA group (2209 ± 408 mm^3^ *vs* 2384 ± 398 mm^3^, *p* = 0.249, Fig. 2b). Both sides of hippocampal volume are positively correlated with recognition scores (Fig. 3a,b) and short-time recall scores (Fig. 3c,d) in both groups.

### Cortical GM volume loss in PCA and tAD patients

**Figure 4:**
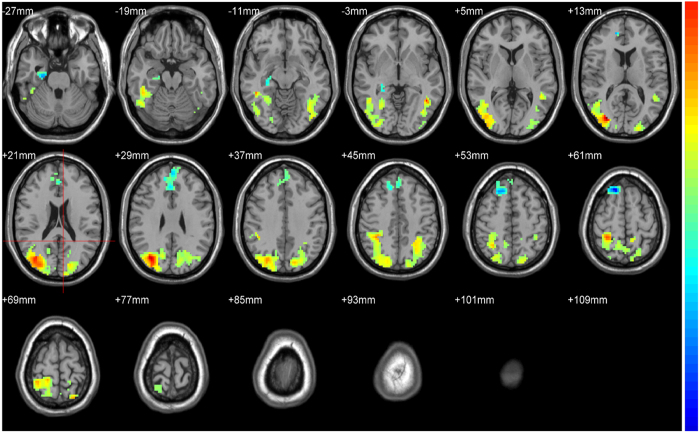
The gray matter (GM) volume loss in PCA group versus tAD group. The t maps indicate the significant differences between patient groups, and the significantly reduced GM volume in the PCA group (red and yellow parts) and in the tAD group (blue parts) were shown. All maps were thresholded at voxel-level with FDR-corrected *p* < 0.05, and the axial slices were shown.

**Figure 5:**
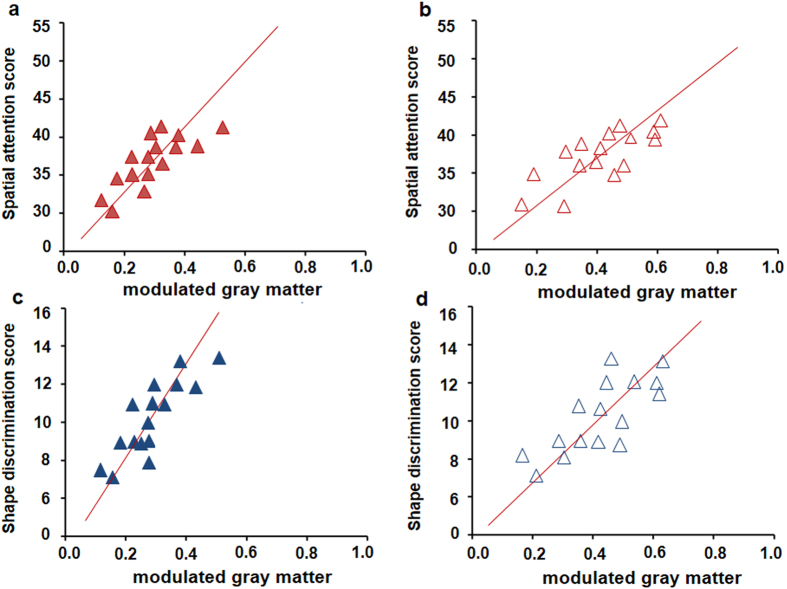
The correlation between the neurocognitive scores and the gray matter (GM) volume of parietal and occipital lobes in PCA patients. (**a**) Scatterplot of individual performance on spatial attention test and mean GM volume of parietal lobe. (**b**) Scatterplot of individual performance on spatial attention test and mean GM volume of occipital lobe. (**c**) Scatterplot of individual performance on shape discrimination test and mean GM volume of parietal lobe. (**d**) Scatterplot of individual performance on shape discrimination test and mean GM volume of occipital lobe. Using the Spearman correlation test with the significant difference of *p* < 0.05.

Compared with healthy controls, PCA patients exhibited significant GM volumes loss in medial temporal, right lateral occipitoparietal, occipital, and partial parietal lobes, whereas tAD patients showed significant GM atrophy mainly in temporal, partial parietal lobes and posterior cingulate cortex (Supplementary Figure 1). Compared with the tAD group, the PCA patients showed significantly GM volume loss in bilateral occipital and parietal lobes, and also the right occipitoparietal and occipitotemporal junctions (Fig. 4). Moreover, tAD patients exhibited significantly GM volumes loss, predominantly over the bilateral inferior frontal lobules and right hippocampus (Fig. 4). Whereas in PCA patients, ROI-based correlation analysis showed positive relationships between spatial attention scores and cortical GM volume in the parietal lobe (*r* = 0.68, *p* = 0.024, Fig. 5a) and occipital lobe (*r* = 0.53, *p* = 0.040, Fig. 5b). Shape discrimination scores were also significantly correlated with modulated GM volume in both the parietal lobe (*r* = 0.82, *p* = 0.011, Fig. 5c) and occipital lobe (*r* = 0.57, *p* = 0.046, Fig. 5d). While calculation scores are significantly correlated with modulated GM volume in only the parietal lobe (*r* = 0.64, *p* = 0.033). There was no significant association between writing score and GM volume in any of the four ROIs (data not shown).

### Regional hypometabolism differences in PCA and tAD patients

**Figure 6:**
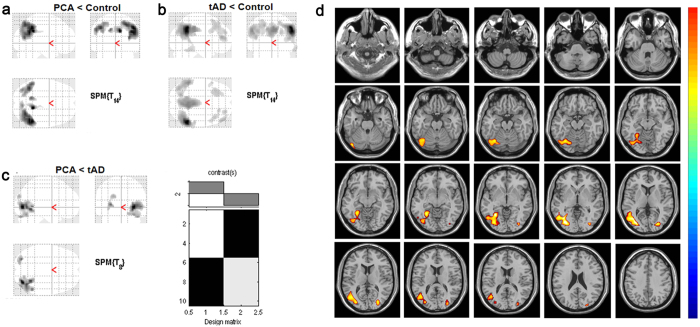
The comparisons of regional hypometabolism between subject groups. (**a**) Compared with healthy controls, the brain regions of significant hypometabolism were shown in PCA patients. (**b**) Compared with healthy controls, the brain regions of significant hypometabolism were shown in tAD patients. (**c**) Compared with tAD patients, the brain regions of significant hypometabolism were shown in PCA patients. The SPM is displayed on axial, coronal and sagittal sections of the custom template. (**d**) The t maps indicate the brain regions with significant hypometabolism in the PCA patients compare to that in the tAD patients, with *p* < 0.001, uncorrected.

Global and regional ^18^F-AV45 or Pittsburg compound uptake are very similar in PCA and AD groups^25^,^26^, therefore we only compared glucose metabolism between these groups. Compared with healthy controls, PCA patients had significant hypometabolism in the precuneus, inferior parietal lobe, superior parietal lobe, occipitotemporal junction, and occipital lobe (Fig. 6a). In tAD patients, significant hypometabolism was found in the lateral frontal lobe, medial temporal lobe, precuneus, occipitoparietal junction, and cingulate gyrus (Fig. 6b). Compared with tAD patients, PCA patients had comparatively significant hypometabolism in the bilateral occipital and inferior parietal lobes, and also the right occipitoparietal and occipitotemporal junctions (Fig. 6c,d). However, no region of significant hypometabolism was found in tAD patients compared with PCA patients. Additionally, out of six ROIs, rCMg of the parietal lobe (including the inferior and superior parietal lobules) and occipital lobe positively correlated with spatial attention and shape discrimination scores in PCA patients (Table 4). In contrast, rCMg of these six ROIs did not show any significant correlations with calculation and writing scores in the PCA group (data not shown).

## Discussion

The present study compared the clinical manifestations, neuropsychological performances, cerebral atrophy, and glucose metabolism between the PCA and tAD patients in a Chinese population. All patients had disease durations less than 4 years, with mild to moderate dementia states. The PCA group showed more impairment on visuospatial tasks but performed better in recognition and recall tests. PCA patients showed larger right hippocampal volume compared with tAD patients. Moreover, GM volume of bilateral parietal and occipital lobes was smaller in PCA patients, and the significant hypometabolism was observed in bilateral parietal and occipital lobes, particularly the right occipitotemporal junction in PCA group. Additionally, significant positive correlations were found between the visuospatial tasks scores and the GM volume or rCMg of parietal and occipital lobes in PCA patients. Our study provided a primary view of neuropsychological and neuroimaging characteristics between PCA and tAD patients at an early disease stage.

Consistent with an earlier report^27^, our PCA and tAD subgroups showed some distinctive clinical signs and cognitive performances. As described previously^28^, compared with tAD patients, PCA patients had more severe symptoms related to visuospatial and visuoperceptual abilities, and they showed greater impairments in writing, calculation, spatial attention, and shape discrimination. In contrast, tAD patients showed greater short-term memory and recall impairments. Moreover, in agreement with a recent study^29^, PCA patients had significantly higher anxiety scores than tAD patients, suggesting they may have insight into their condition. Since we used the same inclusion criteria, it is possible that the PCA patients in our study present some similar clinical symptoms and neuropsychological performances as that in most studies did in Western populations. However, we found a much higher proportion of PCA patients with parietal lobe dysfunction, while the temporal lobe-related functions of naming and verbal fluency were comparable between PCA and tAD patients.

Some studies report no *APOE* genotype differences^30^,^31^, while another showed a significant difference in *APOE4* allele frequency between PCA and tAD patients^32^. We found no significant difference in *APOE* ε4 allele frequency between PCA and tAD groups. Discrepancies between different studies may reflect differences in inclusion criteria and the number of subjects enrolled. Furthermore, an earlier study reported similar levels of biomarkers, including Aβ42, t-tau, and p-tau181, in CSF of PCA and tAD patients^10^. Other studies also found similar levels of t-tau and p-tau in tAD and PCA patients^32^,^33^ (but see^34^). Here, we found similar Aβ42, t-tau, and p-tau levels in CSF and serum of both patient groups, supporting the conclusion that neuropathological changes in PCA are similar to that of amnestic tAD.

Compared with healthy controls, PCA patients always show significant atrophy, primarily in the right parietal and bilateral posterior parietal regions, but also in the occipital lobe. In contrast, tAD patients show greater atrophy predominantly in the hippocampi and left medial temporal lobe^22^,^35^,^36^. Thus, our results are comparable to earlier findings in non-Chinese populations. Nonetheless, there are not many clinical cohort studies directly comparing GM volume differences between PCA and tAD patients. Considerable regional overlap in atrophy (including the posterior cingulate gyri, precuneus, and inferior parietal lobe) has been reported in both PCA and tAD patients^37^. And larger GM volume in the left parahippocampal gyrus and hippocampus was reported in PCA compared with tAD patients^22^,^38^. However, we found larger hippocampal volumes in PCA patients compared with tAD patients, which was more evident in the right hippocampus. And notably, tAD patients do show significant GM loss in both the anterior and posterior cingulate compared with healthy controls, but when compared with PCA patients, there is no significant difference of GM loss in these regions. These may be because the tAD patients enrolled in our study are at a comparatively early stage of disease, and our results are in accordance with two earlier studies^15^,^22^. Moreover, GM volume loss was more significant in PCA patients in the bilateral occipital and parietal lobes, and the right occipitoparietal and occipitotemporal junctions. These results are consistent with the observed clinical manifestations. For example, PCA patients show higher levels of visuospatial function impairments, while tAD patients present with greater memory impairments. We also found that memory scores positively associate with hippocampal volumes, while calculation and spatial attention scores significantly associate with GM volume in parietal and occipital lobes. These findings further confirm an association between neural atrophy and cognitive deficits observed in both PCA and tAD patients, which are supported by the earlier findings^39^,^40^. And to the best of our knowledge, this is the first study to investigate the relationships between neuropsychological tests scores and patterns of brain atrophy of PCA variants in Chinese population.

We did not compare plaque tracer uptake but examined glucose metabolism, which correlates with disease progression^7^. An early study demonstrated hypometabolism in bilateral temporoparietal and precuneal regions in tAD patients^41^. Other studies have shown significant hypometabolism in occipital, parietal, and posterior temporal cortices in PCA patients compared with healthy controls^42^,^43^. Our present results are mostly in line with these previous reports^42^,^43^,^44^, indicating severe hypometabolism in PCA patients primarily in the right occipitotemporal junction, but also including the inferior parietal and occipital lobes. In addition to posterior regions, an early FDG-PET study also revealed specific areas of hypometabolism in the frontal eye fields bilaterally^45^, but we did not find these significant changes, which may due to the early disease stage of our PCA patients. With regard to hypometabolism between AD and PCA patients, Lehmann *et al*. found greater occipitoparietal involvement in PCA, and symmetric involvement of temporoparietal regions in tAD patients^9^. Recently, a Chinese research group found significant hypometabolism exclusively in the right occipital lobe of PCA patients compared with tAD patients (but the sample size was small)^19^. Similarly, we found significant hypometabolism in the bilateral occipital and inferior parietal lobes, and right occipitoparietal and occipitotemporal junctions in PCA patients. The small differences between our and other studies may be owing to differences in disease stage, case numbers, and statistical analysis methods. Interestingly, consistent with the findings of other studies^19^,^26^, we found that tAD patients do not show relative hypometabolism compared with PCA patients. Moreover, we also found that the visuospatial-related dysfunctions are associated with the hypometabolism in parietal and occipital cortices of PCA patients, which is not observed for calculation and writing scores. This may be because of the small sample size of our cohort.

There are some limitations in our study. For example, we recruited participants based on the clinical criteria of PCA and amnestic tAD, some of our results are dictated by the inclusion criteria. We enrolled only a relatively small number of subjects. This is largely owing to the occurrence of this rare degenerative disease, and also difficulties in recruitment of a large number of patients at an early disease stage^15^. Additionally, whilst all PCA patients met the clinical criteria for the syndrome, it remains possible that some participants do not have AD, and may instead have coexistent pathologies. Another potential concern is that our results are primarily from ante-mortem imaging, and lack *in vivo* AD pathology confirmation. Additionally, PCA onset is usually at an early age. Thus, to recruit age-matched tAD patients, we used early-onset patients. This may minimize differences in neuroimaging manifestations between tAD and PCA groups. In addition, the limitation inherent to the techniques of normalization and segmentation within VBM can be the problem in the analysis of atrophic brains^46^, whereas this issue is not specific to this study. Therefore, more participants with follow-up amyloid-PET imaging are needed in our future investigations, which would further our understanding of the underlying mechanisms and enable more conclusive distinctions between PCA and tAD patients.

## Conclusions

Overall, our study provides important information regarding the clinical manifestation, patterns of cerebral atrophy, AD biomarker levels, and regional glucose metabolism in PCA and tAD patients at an early disease stage. Compared with tAD patients, PCA patients show more symptoms related to the visual cortex, and exhibit marked impairments in visuospatial-related cognitive function. These changes in neuropsychological performances are likely associated with specific cortical GM volume loss and hypometabolism in the parietal and occipital lobes. Furthermore, we found no differences in *APOE4* allele frequency and Aβ levels in CSF and serum between PCA and tAD patients. The information gained from our study leads to more insights into the neural change patterns and clinical features of PCA patients in Chinese population, which will be important for clinical diagnosis and treatment of PCA.

## Additional Information

**How to cite this article**: Peng, G. *et al*. Clinical and neuroimaging differences between posterior cortical atrophy and typical amnestic Alzheimer’s disease patients at an early disease stage. *Sci. Rep.* **6**, 29372; doi: 10.1038/srep29372 (2016).

## Supplementary Material

Supplementary Figure 1

## Figures and Tables

**Table 1:** The demographic characteristics in three subject groups.

demographic information	PCA (n = 16)	tAD (n = 13)	control (n = 15)	p value
Gender (male : female)	9 : 7	6 : 7	7 : 8	0.765
Age (mean years ± SD)	55.8 ± 6.5	59.9 ± 8.2	57.6 ± 7.3	0.333
Disease duration(mean years ± SD)	2.4 ± 0.9	3.0 ± 1.0	/	0.159
Education level (mean years ± SD)	8.5 ± 2.7	7.9 ± 2.2	9.1 ± 2.5	0.453
CDR rate (mean ± SD)	1.93 ± 0.68	1.92 ± 0.64	0	0.972
Hypertension-%	18.7%	15.4%	20%	0.898
Diabetes mellitus-%	12.5%	15.4%	6.7%	0.757
Hyperlipoidemia-%	25%	23.1%	20%	0.946
Atrial fibrillation-%	6.3%	7.7%	6.7%	0.988
*APOE 4* allele	12.5%	15.4%	6.7%	0.757

*Notes*: CDR, Clinical Dementia Rating Scale; *APOE*, apolipoprotein E.

**Table 2:** The frequency of each clinical symptom in PCA and tAD groups.

Clinical characteristic	PCA (n = 16)	tAD (n = 13)	p value
Balint’s syndrome (any)-%	37.5%	7.70%	0.013
Gerstmann’s syndrome (any)-%	75.0%	23.1%	0.016
Environment disorientation-%	62.5%	30.8%	0.185
Dressing apraxia-%	37.5%	7.70%	0.013
Construction apraxia-%	68.8%	15.4%	0.027
Ideomotor apraxia-%	18.7%	7.70%	0.751
Neglect-%	37.5%	15.4%	0.364
Finger agnosia-%	81.3%	7.70%	0.000
Prosopagnosia-%	37.5%	0%	0.000
Visual hallucinations-%	6.25%	23.1%	0.110
Memory symptoms-%	68.8%	100%	0.085

**Table 3:** The neuropsychological assessment scores in PCA and tAD groups.

Neuropsychology test	Max score	PCA (n = 16)	tAD (n = 13)	p value
Short recognition memory test for word	25	18.9 ± 2.3	15.8 ± 1.4	0.000
Short recognition memory test for face	25	17.5 ± 2.2	16.9 ± 2.3	0.480
Naming (verbal description)	30	16.9 ± 2.5	18.7 ± 2.9	0.084

Calculation	5	0.8 ± 0.7	2.3 ± 0.9	0.000
Digit span (forwards)	12	5.3 ± 1.0	5.7 ± 0.9	0.273
Digit span (backwards)	12	3.4 ± 0.9	4.2 ± 1.7	0.144
Spatial attention (small word)	48	37.4 ± 2.8	44.5 ± 1.3	0.000
Spatial attention (large word)	48	33.1 ± 1.3	45.2 ± 1.5	0.000
Shape discrimination	20	10.4 ± 1.8	17.8 ± 2.7	0.000
Writing test	25	7.4 ± 2.3	16.1 ± 2.0	0.000
Clock draw test (CDT)	4	1.2 ± 0.5	2.0 ± 0.7	0.001
Category Fluency (1 min of animal)	/	9.7 ± 2.0	10.1 ± 1.4	0.548
CVLT 10 min recall	25	13.8 ± 2.3	10.7 ± 1.3	0.000
HAMA-17	68	25.1 ± 3.2	16.7 ± 2.4	0.000
HAMD-17	68	18.1 ± 2.3	18.7 ± 3.2	0.562
MMSE	30	19.1 ± 3.8	18.9 ± 3.5	0.843

*Notes*: CVLT, California verbal learning test; HAMA, Hamilton anxiety scale; HAMD, Hamilton depression scale; MMSE, Mini-Mental State Examination.

**Table 4:** The correlation of rCMg in 6 ROIs with SD and SA scores in PCA.

*SD score*	IP	SP	STG	MFG	PCCP	OL
correlation	0.584	0.669	0.334	0.526	0.432	0.484
p value	0.037	0.024	0.098	0.069	0.115	0.046
*SA score*						
correlation	0.495	0.706	0.504	0.483	0.518	0.556
p value	0.028	0.017	0.129	0.081	0.074	0.039

*Notes*: SD, shape discrimination; SA, spatial attention; IP, inferior parietal lobe; SP, superior parietal lobe; STG, superior temporal gyrus; MFG, middle frontal gyrus; PCCP, posterior cingulate cortex and precuneus; OL: Occipital lobe; rCMg, regional cerebral glucose metabolism; ROIs, regions of interest.
