# Supplementary material for: Clinical and neuroimaging differences between posterior cortical atrophy and typical amnestic Alzheimer’s disease patients at an early disease stage
Source: Sci Rep. 2016 Jul 5;6:29372. doi: 10.1038/srep29372 (PMC4932506; doi:10.1038/srep29372)

**Clinical and neuroimaging differences between posterior cortical atrophy and typical amnestic Alzheimer’s disease patients at an early disease stage**

GuopingPeng^1,4^, Jianqin Wang^1^, Zhan Feng^2^, PingLiu^1^, Yafei Zhang^3^, FangpingHe^4^, Zhongqin Chen^1^, Kui Zhao^3^, BenyanLuo1^,4,*^

**Supplementary Figure Legend**

**Figure 1. The cortical gray matter (GM) volume loss in patients versus controls.** The t maps indicate the significant difference of GM loss between the PCA group and the control group (a), and also the tAD group *vs* the control group (b). The significantly reduced GM volume in the patient groups (red and yellow parts) were shown. All maps were thresholded at cluster-level FDR-corrected *p*<0.05, and only the axial slices were shown.

**Figure 1.**


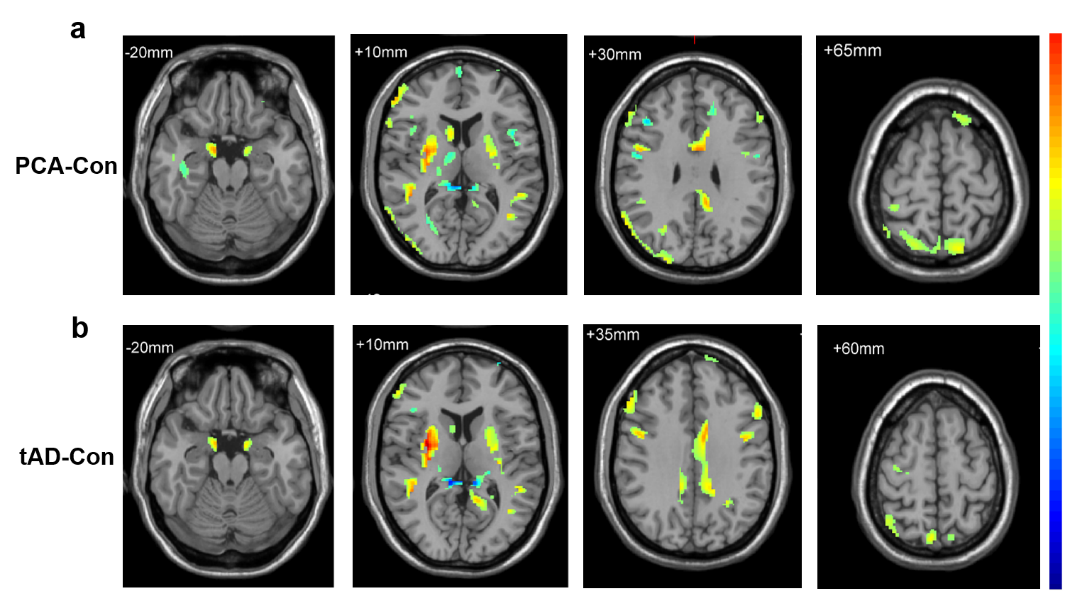

Supplement: Supplementary Figure 1 [file srep29372-s1.doc]
